# Mycobiota and C-Type Lectin Receptors in Cancers: Know thy Neighbors

**DOI:** 10.3389/fmicb.2022.946995

**Published:** 2022-07-13

**Authors:** Lilong Zhang, Dongqi Chai, Chen Chen, Chunlei Li, Zhendong Qiu, Tianrui Kuang, Mungur Parveena, Keshuai Dong, Jia Yu, Wenhong Deng, Weixing Wang

**Affiliations:** ^1^Department of General Surgery, Renmin Hospital of Wuhan University, Wuhan, China; ^2^Hubei Key Laboratory of Digestive System Disease, Wuhan, China

**Keywords:** gut mycobiota, dysbiosis, cancer, CARD9, Dectin-1, Dectin-2, Dectin-3, Mincle

## Abstract

Numerous studies have demonstrated the importance of gut bacteria in the development of malignancy, while relatively little research has been done on gut mycobiota. As a part of the gut microbiome, the percentage of gut mycobiota is negligible compared to gut bacteria. However, the effect of gut fungi on human health and disease is significant. This review systematically summarizes the research progress on mycobiota, especially gut fungi, in patients with head and neck cancer (HNC), esophageal cancer (EC), gastric cancer (GC), colorectal cancer (CRC), hepatocellular carcinoma (HCC), pancreatic cancer, melanoma, breast cancer, and lung carcinoma-induced cachexia. Moreover, we also describe, for the first time in detail, the role of the fungal recognition receptors, C-type lectin receptors (CLRs) (Dectin-1, Dectin-2, Dectin-3, and Mincle) and their downstream effector caspase recruitment domain-containing protein 9 (CARD9), in tumors to provide a reference for further research on intestinal fungi in the diagnosis and treatment of malignant tumors.

## Introduction

Currently, extensive research has been focusing on the impact of the gut microbiome on human health and disease (El-Jurdi and Ghannoum, 2017; Hall and Noverr, 2017; Hoggard et al., 2018). Although the term, “microbiome” implies microorganisms, including bacteria, fungi, protozoa, parasites, and viruses, most studies have concentrated on the intestinal bacteriome (El-Jurdi and Ghannoum, 2017), accounting for over 99% of the gut microbiome (Qin et al., 2010). However, intestinal mycobiota are remarkably larger than intestinal bacteria in cell size and have specialized metabolic gene clusters that respond to specific ecological needs (Wisecaver et al., 2014). They play a vital role in developing and maintaining the human immune system and can be altered in both intestinal and extraintestinal diseases (Coker et al., 2019; Lai et al., 2019). According to the World Health Organization, cancer is the leading cause of death worldwide. In recent years, there has been increasing interest in the potential role of intestinal fungi in the development of human cancers. This study provides a systematic overview of the role of mycobiota, especially gut fungi, and their recognition receptors, C-type lectin receptors (CLRs), to understand the carcinogenic potential of fungi and provide a new theoretical basis for cancer control.

## Gut Mycobiota and Gastrointestinal Neoplasms

The gastrointestinal tract serves as a home for enteric mycobiota, and the relationship between intestinal fungi and gastrointestinal tumors has been extensively studied, particularly in colorectal cancer (CRC). Here, we summarize the role of mycobiota in various gastrointestinal neoplasms.

### Oral Mycobiota and Head and Neck Cancer

**FIGURE 1:**
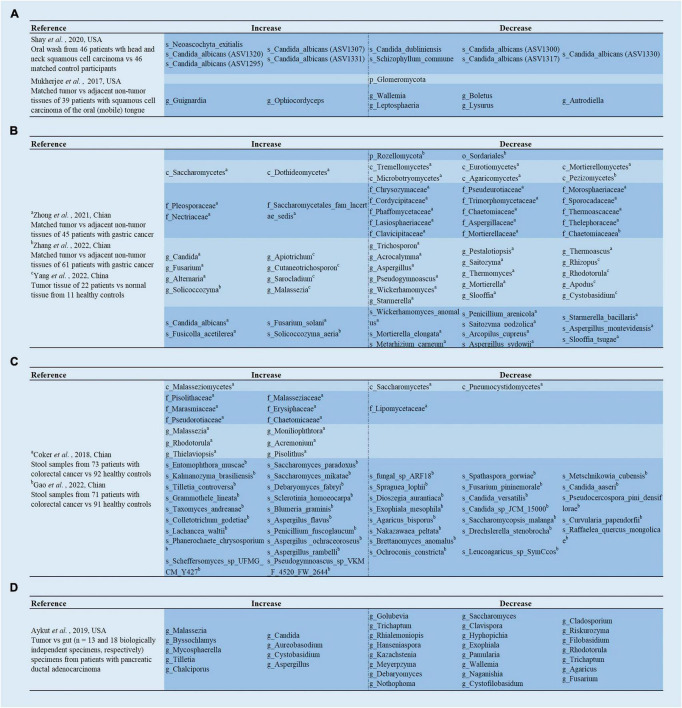
Analysis of the differences in gut mycobiota. **(A)** Shay et al. (2020) demonstrated the differences of the mycobiome in the oral washings from patients with head and neck squamous cell carcinoma (HNSCC) compared to matched control participants; Mukherjee et al. (2017) revealed the differences of the mycobiome in the tumor tissues compared to adjacent non-tumor tissues from patients with oral squamous cell carcinoma OSCC (mobile) in the tongue. **(B)** Zhong et al. (2021) and Zhang et al. (2022) revealed the differences of the mycobiome in the tumor tissues compared to adjacent non-tumor tissues from patients with gastric cancer; Yang et al. (2022) revealed the differences of the mycobiome in the tumor tissues from patients with gastric cancer compared to normal tissue from healthy controls. **(C)** Coker et al. (2019) and Gao et al. (2022) revealed the differences of the mycobiome in the stool samples from patients with colorectal cancer compared to healthy controls. **(D)** Aykut et al. (2019) revealed the differences of the mycobiome in the tumor specimens compared to gut specimens from patients with pancreatic ductal adenocarcinoma.

Head and neck cancer (HNC) arises from epithelial cells and occurs in various anatomical localizations of the upper aerodigestive tract. HNC is the sixth most common cancer worldwide (Johnson et al., 2020). Although treatments have evolved considerably over the years, the quality of life and long-term survival of patients treated for HNC remain poor. Thus, it is crucial to explore the pathogenesis and the means of early diagnosis of HNC to achieve its effective prevention and control. Saliva diagnosis is a fast-evolving field, particularly in HNC. Recently, Mohamed et al. (2021) explored the potential value of the mycobiome in the saliva of patients with oral squamous cell carcinoma (OSCC). Kaplan-Meier survival analysis showed that higher *Candida* carriage was notably related to a shorter overall survival (OS). In contrast, higher salivary *Malassezia* carriage was remarkably correlated with favorable OS in patients with OSCC. The Cox proportional hazards multiple regression model (adjusted for age) revealed that *Malassezia* was an independent predictor of OS (Mohamed et al., 2021). Interestingly, a recent study confirmed that sublingual application of *Candida* or *D-zymosan* significantly accelerated and worsened tongue dysplasia and hyperplasia (Bhaskaran et al., 2021). Moreover, the enrichment of *Candida albicans* has also been correlated with an increase in the inflammatory cytokines interleukin (IL)-1β and IL-8 in the saliva of patients with head and neck squamous cell carcinoma (HNSCC) (Vesty et al., 2018). Fungi in the oral wash of patients with HNSCC were further explored by Shay et al. (2020). They found reduced fungal evenness and richness in the HNSCC oral wash compared to healthy controls. Notably, specific strains of *C. albicans* were over- and under-represented, while *Schizophyllum commune* was depleted in oral wash samples from HNSCC patients (Figure 1A). *S. commune* is known to produce the polysaccharide compound, schizophyllan (Sung et al., 2018), which has anti-tumor properties *in vitro* and shows promise for the treatment of cancers, including HNSCC (Kimura et al., 1994; Mansour et al., 2012; Sung et al., 2018).

**FIGURE 2:**
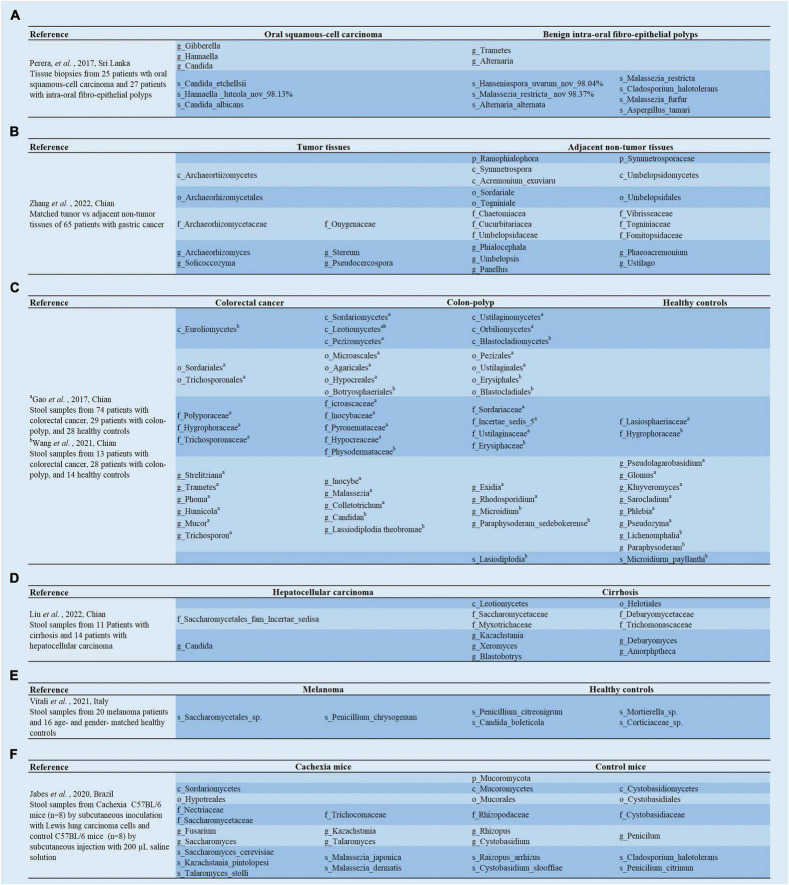
**(A–F)** Analysis of the linear discriminant analysis effect size (LEfSe) in gut mycobiota between cancer patients and healthy controls.

To ensure a relevant correlation with carcinogenesis, deep tissue biopsies are needed instead of saliva or surface swabs. Mukherjee et al. (2017) first observed that fungal richness was remarkably lower in OSCC (mobile) of tongue (OMTC) compared to the adjacent non-tumor tissues (Figure 1A). Notably, they also found a reduced abundance of *Emericella* in tumor tissues. *In vitro* studies have shown that *Emericella* exposure leads to increased p53 expression in colon cancer cell lines, which exerts anti-cancer effects (Mohamed, 2012). Therefore, the reduced abundance of this genus may induce decreased p53 expression, resulting in an oral cancer phenotype. Simultaneously, Perera et al. (2017) explored fungal differences in patients with OSCC and benign intra-oral fibro-epithelial polyps (FEPs). The diversity analysis was consistent with the above study, in patients with OSSC having significantly lower fungal evenness and richness than patients with FEP. Furthermore, compared to FEP populations, an overgrowth of *C. albicans* was detected in the OSCC cohort (Perera et al., 2017; Figure 2A). Interestingly, *C. albicans* was recently found to accelerate OSCC progression *in vitro* by increasing the synthesis of matrix metalloproteinases, oncometabolites, promoting protumor signaling pathways, and the upregulation of prognostic marker genes associated with metastatic events (Vadovics et al., 2022). Thus, *C. albicans* can promote the malignant progression of HNC. Intervention with *C. albicans* might serve as a potential therapeutic route for HNC.

### Mycobiome and Esophageal Cancer

Esophageal cancer (EC) impacts more than 450,000 people worldwide and has the sixth highest mortality rate of all cancers (Sung et al., 2021). Esophageal squamous cell carcinoma (ESCC) and esophageal adenocarcinoma (EAC) are two main subtypes of EC, among which ESCC accounts for 90% of EC cases worldwide (Rustgi and El-Serag, 2014). The role of fungi in EC has not been extensively explored. However, some studies have suggested that specific fungal species may be involved in patients with ESCC having autoimmune polyendocrinopathy-candidiasis-ectodermal dystrophy (APECED) (Rautemaa et al., 2007; Zhu et al., 2017). APECED is a T-cell-driven autoimmune disease caused by mutations in the autoimmune regulator gene that induces defective T-cell central tolerance that hampers the elimination of T-lymphocytes responsive to host cells (Mathis and Benoist, 2009; Xing and Hogquist, 2012). Diminished or defective central tolerance makes patients with APECED more susceptible to developing chronic fungal infections and ESCC (Rautemaa et al., 2007; Manley et al., 2011). Rautemaa et al. (2007) assessed cancer risk in 92 patients with APECED and found that six out of the 92 patients had OSCC or ESCC. Five of these six patients had a history of oral candidiasis. Recently, Zhu et al. (2017) found that the nuclear factor, NF-κB inhibitor kinase alpha [Ikkα, plays a crucial role in forming and maintaining skin homeostasis (Hu et al., 1999; Liu et al., 2008) and functions as a tumor suppressor in the skin (Xia et al., 2010)] of knock-in mice developed phenotypes reminiscent of APECED, including impaired central tolerance, autoreactive T cells, chronic fungal infections, and ESCC. They also demonstrated that autoreactive CD4^+^ T cells permitted fungal infections, incited tissue injury, and inflammation. Antifungal treatment or depletion of autoreactive CD4^+^ T cells inhibited ESCC progression, whereas oral administration of *Cladosporium cladosporioides* promoted ESCC development (Zhu et al., 2017). The authors also revealed similarities between the hallmarks of ESCC from both patients who do not have APECED and Ikkα knockout mice (Zhu et al., 2017). Therefore, chronic fungal infections might promote ESCC, and antifungal therapy might be a potential candidate for the effective prevention and treatment of ESCC.

### Gut Mycobiota and Gastric Cancer

Gastric cancer (GC) is a highly fatal disease with the fifth-highest incidence and fourth highest mortality rate globally (Sung et al., 2021). Most cases are of the intestinal type of non-cardia GC, which often progresses histologically from atrophic gastritis (AG) to intestinal metaplasia and eventually to GC (Park and Kim, 2015). It is known that *Helicobacter pylori* (*H. pylori*) infection is the leading risk factor for this histological alteration, which causes inflammation of the gastric mucosa and disruption of the associated hydrochloric acid-secreting glands, leading to AG (Amieva and Peek, 2016). AG is a chronic inflammatory, hypochloremic state that may induce GC. Although *H. pylori* infection is known to contribute to this cascade, only about 1–3% of infected individuals subsequently develop GC (Peek and Crabtree, 2006; Stewart et al., 2020), suggesting that the presence of other components plays a crucial role in developing GC. The mycobiota were not involved in the carcinogenesis of GC, until recently. Zhong et al. (2021) analyzed the mycobiome of cancer lesions and adjacent non-cancerous tissues from 45 patients with GC. They found that there was a mycobiome disorder in GC tissues compared to paraneoplastic tissues, characterized by altered fungal composition and ecology (Figure 1B). Specifically, compared to the adjacent tissues, the alpha diversity of the mycobiome was significantly reduced in the GC tissues. The principal component analysis also revealed separate clusters between GC and adjacent tissues. More notably, they found that *C. albicans* was significantly elevated in GC tissues and could be used as a fungal biomarker for GC (Zhong et al., 2021). *C. albicans* might be involved in the pathogenesis of GC by reducing the diversity and abundance of fungi in the stomach (Zhong et al., 2021). Besides, Zhong et al. (2021) further acquired 10 healthy samples, and found no significant differences between healthy individuals and adjacent non-cancerous tissues.

Another study was not entirely consistent with these results, as it found no significant difference in alpha and beta diversity between the GC and the matched para-GC groups. However, the results also indicated that compared to gastric tissue from healthy populations, GC tissue had significantly lower alpha diversity of fungi, and the GC group also formed a relatively well-separated fungal flora (Yang et al., 2022). Furthermore, fungal dysbiosis was reflected by a higher proportion of opportunistic fungi, such as *Cutaneotrichosporon* and *Malassezia* in patients with GC compared to healthy controls (Yang et al., 2022; Figure 1B). The receiver operating characteristic (ROC) curves showed great diagnostic potential in distinguishing GC and healthy controls, including *Cystobasidium, Cutaneotrichosporon, Apiotrichum, Simplicillium, Lecanactis, Rhizopus, Rhodotorula, Exophiala*, and *Sarocladium* (Yang et al., 2022). Similar to the above study by Yang et al. (2022), Zhang et al. (2022) discovered no significant difference in fungal diversity between tumor and paracancerous tissues. Furthermore, they demonstrated that *Solicoccozyma* and *Solicoccozyma aeria* were significantly enriched in the tumor tissue (Figures 1B, 2B). In particular, the abundance of these two taxa was significantly higher in patients with GC at stage I or no nerve invasion than in patients with GC at stage II-IV or nerve invasion (Zhang et al., 2022).

### Gut Mycobiome and Colorectal Cancer

According to Sung et al. (2021) CRC is the second most common contributor to cancer death (Sung et al., 2021). Recently, the CRC incidence rate has increased among young adults, and younger CRC patients present with more advanced disease, significantly reducing the quality of life of survivors and increasing the socioeconomic burden (Virostko et al., 2019). More than 80% of sporadic CRC cases are caused by colorectal adenomas (Jass, 2007; Ullman and Itzkowitz, 2011). Colorectal adenomas are classified as advanced or non-advanced (Davila et al., 2006). Advanced adenomas can further progress to carcinoma. With research progression, the link between fecal or mucosal mycobiota, colorectal adenomas, and CRC has received significant attention.

#### Mucosal Mycobiota in Adenoma and Colorectal Cancer

Mucosal mycobiota is more stable than its luminal counterpart due to the adhering capacity of the organisms to the surface-associated polysaccharide matrices of the intestinal epithelium. The study of mucosal fungi is essential for exploring the pathogenesis of CRC. Luan et al. (2015a,b) focused on comparing the mucosal mycobiome in biopsy adenomas and adjacent tissue. The results indicated that two opportunistic fungal pathogens, *Phoma* and *Candida*, represented an average of 45% of the mycobiome. However, the fungi at the phylum, genus, or species level did not show any significant difference between adenomas and adjacent normal biopsy tissue by *t*-test. Operational taxonomic unit-level analysis revealed a decreased diversity in adenomas compared with adjacent tissue and a significant discrepancy between advanced and non-advanced adenoma tissue (Luan et al., 2015b). Chronic intestinal inflammation is a potential risk factor for CRC development in patients with inflammatory bowel disease (IBD). The type of CRC that IBD precedes is colitis-associated cancer (CAC) (Feagins et al., 2009; Saleh and Trinchieri, 2011; Rubin et al., 2012). Richard et al. (2018) analyzed the mucosa-associated fungal constituents among 10 patients with CRC (without IBD), 7 patients with CAC, and 10 healthy individuals, and revealed there was no significant difference. Thus, the current evidence shows no significant differences in the mucosal fungi between adenomas and adjacent normal tissue, and between patients with CRC and healthy individuals.

#### Fecal Mycobiota in Adenoma and Colorectal Cancer

Usually, the ratio of *Basidiomycota*/*Ascomycota* is considered an indicator of fungal dysbiosis (Kuramae et al., 2013; Malik et al., 2016). Gao et al. (2017) first characterized the fecal mycobiota of 74 patients with CRC, 29 patients with polyp, and 28 healthy individuals using ITS sequencing (Figure 2C; Gao et al., 2017). In patients with colon polyps and CRC, fungal dysbiosis was observed, including elevated diversity in patients with polyp than in healthy individuals, an increased *Ascomycota*/*Basidiomycota* ratio, and a higher proportion of opportunistic fungi *Trichosporon* and *Malassezia* in patients with colon polyps and CRC than in healthy controls (Gao et al., 2017). Subsequent analyses regarding tumor stage showed that patients with late-stage CRC possessed a higher diversity of mycobiota and a higher abundance of *Microbotryomycetes, Sordariomycetes, Microascaceae, Sordariales unidentified, Lasiosphaeriaceae unidentified, Sordariales unidentified 1*, and *Microascales*, and a lower abundance of *Pleosporaceae* and *Alternaria* compared to those with early stage CRC (Gao et al., 2017). Next, the team re-analyzed 71 patients with CRC, 63 patients with adenoma, and 91 healthy controls for fecal fungi using metagenomic sequencing (Gao et al., 2022; Figure 1C), and found that there were no significant differences in alpha diversity, beta diversity, and the ratio of *Basidiomycota*/*Ascomycota* among the three groups. *Phanerochaete chrysosporium, Lachancea waltii*, and *Aspergillus rambellii* were dominant in patients with CRC, while *Candida versatilis, Pseudocercospora pini densiflorae*, and *Candida* sp. *JCM 15000* were the top 3 fungi significantly enriched in healthy controls. *Malassezia restricta* increased, while *Leucoagaricus* sp. *SymCcos* and *fungal* sp. *ARF18* significantly decreased in patients with adenoma compared to healthy controls (Gao et al., 2022). Similarly, Wang et al. (2021) also estimated differences in intestinal fungi between 24 patients with CRC, 31 patients with colorectal polyps, and 18 normal controls using intergenic spacer (ITS) sequencing (Figure 2C). The results show that the CRC, colorectal polyps, and control groups were clustered separately. The richness of the intestinal fungi in the CRC group was significantly higher than in the colorectal polyp group and control group (Wang et al., 2021). More importantly, they also found a significant increase in *C. albicans* in patients with CRC. Mechanistic studies revealed that by interacting with Dectin-1 in the intestinal epithelial cell (IEC), *C. albicans* promoted IEC proliferation in a Dectin-1- and Wnt-dependent manner (Wang et al., 2021). Notably, the Wnt signaling pathway has been related to cell proliferation, differentiation, apoptosis, motility, and polarization in invertebrates and mammals. Aberrant activation of the Wnt pathway is associated with various cancers (Katoh and Katoh, 2005; Li et al., 2006).

Furthermore, CRC-associated fungal dysbiosis was also identified by a multicenter study comprising patients with CRC and healthy controls (Coker et al., 2019). They revealed separate clusters for CRC and healthy controls, while alpha diversity analysis did not find remarkable differences. Six fungal genera were enriched in the patients with CRC, including *Malassezia, Moniliophthora, Rhodotorula, Acremonium, Thielaviopsis*, and *Pisolithus* (Coker et al., 2019; Figure 1C). The pathogenic potential of these genera in the disease has been identified in different studies. For instance, isolates of *Acremonium* have been recovered from human fluids, and are considered opportunistic pathogens (Perdomo et al., 2011). *Rhodotorula* is an emerging pathogen capable of colonizing, infecting, and destroying the basic microbiota of the human digestive system (Wirth and Goldani, 2012). Interestingly, the ecological analysis by Coker et al. (2019) revealed a higher number of co-occurring fungal intra-kingdom and co-exclusive bacterial-fungal correlations in CRC compared to control, indicating that synergistic ecological associations between them might play a role in colorectal carcinogenesis. However, the *Ascomycota*/*Basidiomycota* ratio was significantly lower in the CRC group than in the control group (Coker et al., 2019), contrary to the previous study by Gao et al. (2017). Notably, crossover studies showed significant variability, which might be due to inconsistent methodology or lack of assessment criteria. Nevertheless, we can still conclude that there was no significant difference in alpha diversity between CRC and healthy controls, while the abundance of opportunistic fungi is significantly higher in CRC.

#### The Potential of Mycobiota for Non-invasive Detection of Colorectal Cancer

Not only do many gut fungi contribute to the development of CRC, but alterations in fungi may also be used to diagnose CRC. Coker et al. (2019) found the abundance of 14 fungal biomarkers (including the rising *Aspergillus flavus, Kwoniella mangrovensis, Pseudogymnoascus* sp. *VKM F-4518, Debaryomyces fabryi, Aspergillus sydowii, Moniliophthora perniciosa, Kwoniella heavenensis, Aspergillus ochraceoroseus, Talaromyces islandicus, Malassezia globosa, Pseudogymnoascus* sp. *VKM F-4520*, and *Aspergillus rambellii* and the falling *Pneumocystis murina* and *Nosema apis*) distinguished CRC from controls. Independent Chinese and European cohorts validated these findings, respectively (Coker et al., 2019). The model constructed by Gao et al. (2022) using 13 fungal species (*Taxomyces andreanae, Aspergillus rambellii, Lachancea waltii, fungal* sp. *ARF18, Phanerochaete chrysosporium, Aspergillus flavus, Fusarium pininemorale, Raffaelea quercus mongolicae, Tilletia controversa, Candida versatilis, Exophiala mesophila, Pseudocercospora pini densiflorae*, and *Brettanomyces anomalus*) also displayed strong diagnostic performance. Notably, a recent study using a CRC metagenomic dataset, analyzing 1,368 samples from 8 distinct geographical cohorts, also showed an excellent diagnostic value of gut fungi for CRC (Liu N. N. et al., 2022). Therefore, the study of gut mycobiome offers new ideas for diagnosing CRC.

### Gut Mycobiota and Hepatocellular Carcinoma

Primary liver cancer is the sixth most common cancer and the third major cause of cancer-related mortality globally, with hepatocellular carcinoma (HCC) comprising 75–85% of cases (Sung et al., 2021). Recently, a study showed significantly lower alpha diversity in patients with HCC than in patients with cirrhotic patients using ITS sequencing on stool samples from 11 cirrhotic patients and 17 HCC patients (Figure 2D; Liu Z. et al., 2022). The results of principal coordinates analysis revealed aggregation in patients with HCC and cirrhosis, respectively. More importantly, it was found that *C. albicans* was significantly enriched in the patients with HCC. The gavage of *C. albicans* in the wild-type mice with HCC increased the tumor size and weight (Liu Z. et al., 2022). Remarkably, the colonization of *C. albicans* did not affect tumor growth in nucleotide oligomerization domain-like receptor family pyrin domain containing 6 (NLRP6) knockout mice (Liu Z. et al., 2022). Thus, intestinal *C. albicans* could promote hepatocarcinogenesis through upregulation of NLRP6.

### Gut Mycobiota and Pancreatic Cancer

Pancreatic cancer is a highly malignant tumor with a 5-year survival rate of only 9% and is known as the “king of cancers” in oncology (Sung et al., 2021). This poor survival rate is due to factors, such as non-specific symptoms, lack of early diagnostic markers, aggressive tumor biology, and resistance to most currently available treatments. There is an urgent need for new prevention, screening, and treatment methods (Giovannetti et al., 2017). Recently, Aykut et al. (2019) pioneered the discovery that fungi can migrate from the intestinal lumen to the pancreas and those pathogenic fungi can promote pancreatic ductal adenocarcinoma (PDA) by activating the mannose-binding lectin (MBL)-complement-3 (C3) pathway. Specifically, PDA tumors display an approximately 3,000-fold increase in fungi compared to normal pancreatic tissue in both human and mouse models. The composition of fungi in PDA tumors was distinct from that of the intestine or normal pancreas. More strikingly, in mice and humans, the mycobiome infiltrating the PDA tumors was significantly enriched in *Malassezia* (Figure 1D). In contrast, benign pancreatic inflammation did not increase fungal infiltration of the pancreas (Aykut et al., 2019). To determine the effect of mycobiome dysbiosis on the progression of PDA, the investigators fed amphotericin B orally to slowly progressing and aggressive models of PDA to ablate their mycobiome, and the results showed that tumor growth was significantly inhibited in both models (Aykut et al., 2019). For patients with PDA, gemcitabine is still among the most widely prescribed drugs, but the response to this treatment is extremely poor (Meijer et al., 2019). Interestingly, the reduction of fungi by antifungal agents enhanced the effect of gemcitabine. However, anti-fungal treatment combined with gemcitabine did not prevent germ-free mouse tumor growth (Aykut et al., 2019). Surprisingly, the repopulation with a *Malassezia* (but not in *Candida* sp. *Saccharomyces cerevisiae*, or *Aspergillus* sp.) accelerated oncogenesis. Finally, it was observed that the MBL binding to glycans of the fungal wall to activate the complement cascade reaction was required for carcinogenic progression (Aykut et al., 2019). As we know, complement activation has many roles, including stimulating cell growth, survival, and migration, all of which are factors that promote tumor growth (Mamidi et al., 2017; Kochanek et al., 2018; Okrój and Potempa, 2018).

Another recent and important study found that intra-tumoral fungi (*Malassezia globosa* or *Alternaria alternata*) enhanced PDA cancer cell secretion of IL-33 by Dectin-1-mediated activation of the src proto-oncogene, non-receptor tyrosine kinase (Src)-spleen tyrosine kinase (Syk)-caspase recruitment domain-containing protein 9 (CARD9)-NF-κB pathway (Alam et al., 2022). The secreted IL-33 recruited and activated Th2 and innate lymphoid cells 2 (ILC2) in the tumor microenvironment (TME), which stimulated tumor growth by secreting pro-tumor cytokines, such as IL-4, IL-5, and IL-13 (Chevalier et al., 2017; Alam et al., 2022). Correspondingly, amphotericin B treatment or IL-33 deficiency significantly reduced tumor burden, improved survival, and decreased tumor-infiltrating ILC2 cells and Th2 cells (Alam et al., 2022). Therefore, it is likely that altering the mycobiome by targeting specific populations could help ameliorate PDA, and anti-fungal treatment might appear to be an attractive therapeutic option for PDA. Alternatively, treatments targeting the immune components, such as MBL and IL-33 that control fungal infections could also provide a promising way to fight this deadly cancer.

## Gut Mycobiota and Non-Gastrointestinal Neoplasms

Although gut fungi reside in the gastrointestinal tract and are closely associated with gastrointestinal tumors, they also play an essential role in the systemic immune response. As a result, their role in non-gastrointestinal tumors is gradually being discovered.

### Gut Mycobiota and Melanoma

Melanoma is an aggressive malignancy caused by the uncontrolled proliferation of abnormal melanocytes. The progress of melanoma is rapid, and its prognosis is very poor, with a 5-year survival rate of 20% (Long et al., 2016; Hamid et al., 2019). The composition of the gut microbiome is associated with the prognosis and evolution of advanced melanoma and is considered a biomarker for immune checkpoint therapy (Gopalakrishnan et al., 2018; Mohiuddin et al., 2021). Recently, the intestinal fungal composition of melanoma patients was investigated for the first time by Vitali et al. (2021). They found significantly higher fungal richness in melanoma patients, and *Saccharomycetales* sp. was enriched in patients with melanoma compared to healthy controls (Figure 2E). Moreover, *Malasseziales* sp. and *Malassezia globosa* were significantly enriched in invasive melanoma compared to *in situ* melanoma (Vitali et al., 2021). Further research also found that the gut fungal profile may be relevant to melanoma regression. Regression involves T lymphocytes recognizing specific melanoma antigens that can destroy melanoma cells, induce progressive (partial or complete) tumor disappearance, and replace them with immune/fibrotic infiltration (Aung et al., 2017). However, its prognostic role remains controversial as the occurrence of immune/fibrotic infiltrates may cause an underestimation of Breslow thickness (Aung et al., 2017; Cartron et al., 2021).

### Gut Mycobiota and Breast Cancer

Breast cancer surpassed lung cancer as the most common cancer worldwide in 2020, accounting for 11.7% of all cancer cases, and it is the fifth most frequent cause of death worldwide (Sung et al., 2021). Over the past decades, considerable progress has been made in diagnosing and treating breast cancer. However, more research is needed to improve the prognosis and overcome the resistance to advanced breast cancer (Ginsburg et al., 2017). Shiao et al. (2021) discovered the modulation of tumor immunity by fecal mycobiota during breast cancer radiotherapy, providing a new idea to improve the clinical efficacy of radiotherapy. They administered antifungal (AF) drugs orally to mice with breast cancer, which had a limited influence on bacterial levels, but reduced gut fungal levels. The results showed that AF combined with radiation therapy (RT) significantly inhibited tumor growth, promoted tumor cell death, and prolonged survival time in mice compared to RT alone (Shiao et al., 2021). Similar efficacy was found in the melanoma model (Shiao et al., 2021). Further examination of the changes in immune cells in tumor tissues showed that CD4^+^ T cells and CD206^+^F4/80^+^ immunosuppressive macrophages were decreased, while CD8^+^ anti-tumor T cells were increased in the AF combined with RT compared to RT alone, suggesting that gut fungi can modulate the tumor immune microenvironment by affecting macrophages and T cells (Shiao et al., 2021). Next, by supplementing *C. albicans* combined with RT in mice with breast cancer, the authors found that the tumor growth was faster, and the survival time was shorter than the RT-only treatment, and the ratio of PD-1^+^CD8^+^ T cells was significantly increased, indicating a more immunosuppressive TME allowing for tumor growth. All of these effects were reversed when *C. albicans* were inhibited with AF (Shiao et al., 2021). Finally, it was discovered that elevated intra-tumoral Dectin-1 expression was negatively related to the survival of patients with breast cancer and was required for the effect of commensal fungi’ on mouse models with RT (Shiao et al., 2021). Previous studies have also reported that *C. albicans* infection can increase Treg numbers in the TME and splenocytes and promote breast cancer growth (Ahmadi et al., 2019). Considering that most patients with cancer are usually on antibiotics, the knowledge that the overgrowth of specific fungi may lead to the creation of an immunosuppressive TME, which reduces the efficiency of anti-tumor therapy, could be very relevant (Riquelme and McAllister, 2021). Thus, intestinal fungi may be an effective target for improving the efficacy of cancer therapy.

### Gut Mycobiota and Lung Carcinoma-Induced Cachexia

Cancer cachexia (CC) is a metabolic syndrome related to several underlying diseases, such as cancer and chronic kidney disease, among many others (Fearon et al., 2011). It is characterized by a decrease in muscle mass, the depletion of body fat, and widespread chronic inflammation (Fearon et al., 2011). Its prevalence in patients with different types of cancer significantly reduces their life quality and expectancy (Bruera and Hui, 2012). Recently, Jabes et al. (2020) explored the alterations of gut fungi in CC mice inducted by subcutaneous inoculation with Lewis lung carcinoma cells (a model widely employed for the study of CC) (Bennani-Baiti and Walsh, 2011). They found that there were sufficient differences in the composition of intestinal fungi to distinguish the CC mice and control mice by beta diversity analysis. Furthermore, linear discriminant analysis effect size (LEfSe) and real-time quantitative PCR showed a significantly higher abundance of *Saccharomyces, Kazachstania, Saccharomyces cerevisiae, Malassezia, Malassezia dermatitis*, and *Malassezia japonica* in the feces of CC mice, while the abundance of *Rhizopus oryzae* was significantly higher in the control mice (Figure 2F; Jabes et al., 2020). Previous studies have shown that *R. oryzae* can produce large amounts of various antioxidants and organic acids, including gallic acid (Londoño-Hernández et al., 2017). Interestingly, an extract of oil palm phenolics, containing 1,500 ppm gallic acid equivalent, has been shown to suppress tumorigenesis, by mediating G1/S phase cell cycle arrest, delaying inflammatory responses, and attenuating CC symptoms in tumor-bearing mice (Leow et al., 2013). Gallic acid has also been found to increase glucose tolerance and triglyceride concentrations in obese mice (Leow et al., 2013), inhibit lipogenesis in humans, and counteract pro-inflammatory responses (Dludla et al., 2018). Thus, there is a disorder of the fecal mycobiome in CC, and *R. oryzae* might be a promising candidate probiotic that deserves further study. However, CC is triggered by different factors that influence the development and outcomes of the syndrome in different ways and might affect the alterations in the gut mycobiota, which needs further exploration.

## The Role of C-Type Lectin Receptors in Cancers

Fungal cell walls are mainly composed of multiple layers of carbohydrates, including mannose polymers (mannans), D-glucose linked by β-glycosidic bonds polymers (β-glucan), and N-acetyl-D-glucosamine polymers (chitins). These cell wall components are believed to be recognized by different CLRs, including Dectin-1 (CLEC7A, CLECSF12), Dectin-2 (CLEC6A, CLEC4N, and CLECSF10), Dectin-3 (CLEC6, CLEC4D, CLECSF8, and MCL), Mincle (CLEC4E, CLECSF9), DC-SIGN (CLEC4L), DCIR (CLEC4A, CLECSF6), etc. Over the past 20 years, CLRs have been shown to play a key role in initiating the host immune response against fungal infection (Brown, 2011; Wüthrich et al., 2012; Wevers et al., 2013; Dambuza and Brown, 2015; Underhill and Pearlman, 2015). CLRs can be classified into four broad categories based on their signaling motifs (Sancho and Reis e Sousa, 2012; Del Fresno et al., 2018; Drouin et al., 2020). Immunoreceptor tyrosine-based activating motif (ITAM)-coupled CLRs either have an ITAM motif constituted by YxxL tandem repeats in their cytoplastic tail, or interact with ITAM-containing adaptors, such as Dectin-2, Dectin-3, and Mincle (Yamasaki et al., 2008; Kerrigan and Brown, 2011; Sancho and Reis e Sousa, 2012; Drouin et al., 2020; Li et al., 2022). Hemi-ITAM-(hemITAM)-bearing CLRs only contains a single tyrosine within their YxxL motif, such as Dectin-1 (Sancho and Reis e Sousa, 2012; Drouin et al., 2020; Li et al., 2022). Activation of these receptors induces intracellular signaling *via* Syk-dependent and independent pathways; the former has been more thoroughly studied. Activation of Syk *via* CLRs triggers the CARD9-B-cell leukemia-lymphoma 10 (BCL10)-mucosa-associated-lymphoid-tissue lymphoma-translocation gene 1 (MALT1) complex-dependent NF-κB signaling pathway (Bi et al., 2010; Gorjestani et al., 2011, 2012), which affects many aspects of both innate and adaptive immunity (Drummond et al., 2011; Kerrigan and Brown, 2011). Unlike ITAM or hemITAM-containing CLRs, immunoreceptor tyrosine-based inhibitory motif (ITIM)-containing CLRs, such as DCIR, negatively regulate the signaling pathway of other pattern recognition receptors (PRRs) (Sancho and Reis e Sousa, 2012; Li et al., 2022). The final category is ITAM-ITIM independent CLRs, such as DC-SIGN, which lack the typical ITAM or ITIM signaling motifs (Li et al., 2022). As previously mentioned, fungi might be involved in cancer development, and recent studies confirm that CLRs, as key recognition receptors for fungal immune responses, are also closely associated with cancer. Thus, we review recent developments in the study of Dectin-1, Dectin-2, Dectin-3, Mincle, and their downstream CARD9 in cancer progression.

### The Pro- and Anti-cancer Roles of Dectin-1 in Cancers

Dectin-1, also known as the β-glucan receptor, is an emerging pattern recognition receptor encoded by C-type lectin domain family 7 member A (CLEC7A) (Huysamen and Brown, 2009; Mayer et al., 2017). β-glucans are the most common pathogen-associated molecular patterns (PAMPs) found in fungal cell walls that are recognized by Dectin-1. The expression of Dectin-1 has been predominantly observed in myeloid cells, including macrophages, dendritic cells (DCs), monocytes, and neutrophils (Brown and Crocker, 2016). The most widely known role of Dectin-1 is antifungal defense, as summarized in these reviews (Kimberg and Brown, 2008; Marakalala et al., 2011; Saijo and Iwakura, 2011). The critical role of Dectin-1 in immune defense provides a general reflection that its deficiency will increase the risk of infectious diseases (Kalia et al., 2021). Dectin-1 knockout mice significantly increase the fungal burden and low survival rates (Taylor et al., 2007).

**FIGURE 3:**
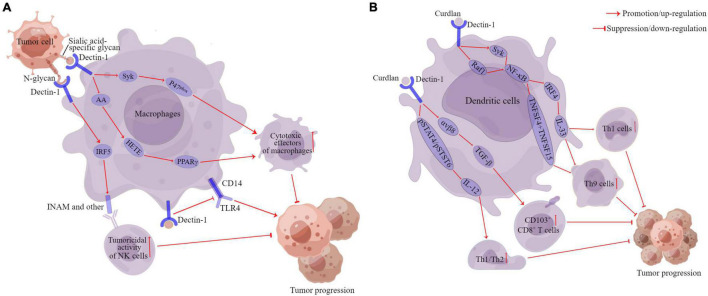
The anti-cancer of Dectin-1 in cancers. Pathways of action of Dectin-1 in macrophages **(A)**. Pathways of action of Dectin-1 in dendritic cells **(B)**. DC, dendritic cells; AA, arachidonic acid; Syk, spleen tyrosine kinase; P47^phox^, neutrophil cytosolic factor 1; HETE, the 12- and 15-hydroxyeicosatrienoic acids; PPARγ, peroxisome proliferator-activated receptor-gamma; IRF5, interferon regulatory factor 5; INAM, family with sequence similarity 26 member F; TLR4, Toll-like receptor 4; Raf1, raf-1 proto-oncogene, serine/threonine kinase; IRF4, interferon regulatory factor 4; IL, interleukin; TNFSF15, tumor necrosis factor ligand superfamily member 15; TNFSF4, tumor necrosis factor ligand superfamily member 4; TGF-β, transforming growth factor-beta 1. αvβ8, αvβ8 integrin pSTAT4, phosphorylation of signal transducer and activator of transcription 4; pSTAT6, phosphorylation of signal transducer and activator of transcription 6. This figure was created using Figdraw (www.figdraw.com).

Along with the concern about the carcinogenic role of fungi, the role of Dectin-1 in tumor development has also attracted the interest of scholars globally. Recently, several studies have demonstrated that Dectin-1 plays an extremely important role in the anti-tumor immune response (Figure 3). For example, Seifert et al. (2015) found that the expression level of Dectin-1 was highly upregulated in both liver fibrosis and HCC (highly expressed in DCs and macrophages, not in hepatocytes). Dectin-1 deficiency accelerated liver fibrosis and hepatocellular tumorigenesis (Seifert et al., 2015). Whereas Dectin-1 activation inhibited Toll-like receptor 4 (TLR4) signaling in hepatic inflammatory and stellate cells by attenuating the TLR4 and its co-receptor CD14 expression, thereby suppressing liver fibrosis and hepatocarcinogenesis (Figure 3A; Seifert et al., 2015). Chiba et al. (2014) indicated that Dectin-1 expression on DCs and macrophages was essential for the enhancement of NK-mediated killing of tumor cells that express high levels of N-glycan structures. Dectin-1 recognition of N-glycan led to the activation of the interferon regulatory factor 5 (IRF5) and downstream genes, such as INAM (termed as a family with sequence similarity 26 member F), inducing the full-blown tumoricidal activity of natural killer (NK) cells (Figure 3A; Chiba et al., 2014). Similarly, a recent study found that IL-13-mediated Dectin-1 and mannose receptor (MR) overexpression promoted the anti-tumor activity of macrophage by recognizing sialic acid-specific glycan structures on the tumor surface. Specifically, Dectin-1 and MR enhanced the cytotoxic function of macrophages through the activation of downstream Syk-neutrophil cytosolic factor 1 (P47^phox^) signaling and arachidonic acid (AA)-the 12- and 15-hydroxyeicosatrienoic acids (HETE)-peroxisome proliferator-activated receptor gamma (PPARγ) axis, thereby inhibiting the progression of T-cell lymphoma, ovarian adenocarcinoma, and breast adenocarcinoma (Figure 3A; Alaeddine et al., 2019).

In addition, Dectin-1 also plays an important role in regulating the anti-tumor effects of DCs. For example, tumor-associated DCs are also activated by Dectin-1, which simultaneously blocks Th2 cells and induces CD103^+^CD8^+^ mucosal T-cell differentiation to eliminate pre-existing breast cancers (Figure 3B; Wu et al., 2014). Th9 cells have been shown to mediate effective anti-tumor effects *in vivo* (Purwar et al., 2012; Schanz et al., 2021). Dectin-1 activated DCs triggered effective anti-tumor immunity by inducing Th9 cells (Figure 3B; Zhao et al., 2016). Specifically, Dectin-1 activated Syk/raf-1 proto-oncogene, serine/threonine kinase (Raf1)-NF-κB signaling pathways in DCs, leading to increased tumor necrosis factor ligand superfamily member 15 (TNFSF15) and tumor necrosis factor ligand superfamily member 4 (TNFSF4) expression, which in turn was instrumental in triggering the differentiation of naive CD4^+^ T cells to Th9 cells (Figure 3B; Zhao et al., 2016). Wang D. et al. (2018) also found that Dectin-1 activation enhanced interferon regulatory factor 4 (IRF4) expression *via* the Syk/Raf1-NF-κB pathways, which consequently upregulated IL-33 expression in DCs. The blockade of IL-33 can inhibit Dectin-1-activated DC-induced Th9 cell differentiation, and the complement of IL-33 further promotes Th1 cell and Th9 cell priming and antitumor efficacy (Figure 3B; Chen et al., 2018).

However, in a contrasting role, some studies have shown that Dectin-1 is an oncogenic marker (Xia et al., 2016; Daley et al., 2017). For example, Xia et al. (2016) found that Dectin-1 was mainly expressed in the tumor cells and that high Dectin-1 expression was an independent predictor of poor clinical outcomes in patients with clear cell renal cell carcinoma (ccRCC). Daley et al. (2017) also revealed upregulated Dectin-1 expression in the tumor and peritumoral inflammatory compartment in PDA, and Dectin-1 signaling accelerated PDA progression. Interestingly, activation or knockdown of Dectin-1 did not affect the proliferative capacity of transformed pancreatic epithelial cells, and the deletion of Dectin-1 in the extra-epithelial compartment was protective against tumorigenesis (Daley et al., 2017). Mechanistic studies revealed that Dectin-1 ligated the lectin Galectin-9 in the PDA TME, leading to tolerogenic macrophage programming and adaptive immunosuppression. Following the disruption of the Dectin-1-Galectin-9 axis, CD4^+^ and CD8^+^ T cells were reprogrammed as indispensable mediators of anti-tumor immunity (Daley et al., 2017). Additionally, it was demonstrated that increased fungal abundance and the resulting aberrant Dectin-1 signaling might play a role in accelerated oral carcinogenesis in aged mice (Bhaskaran et al., 2021). Dectin-1-deficient mice showed reduced infiltration of Tregs and myeloid-derived suppressor cells (MDSCs) in the tongue, and slower tumor progression (Bhaskaran et al., 2021).

All of these factors suggest an important but uncertain role of Dectin-1 in cancer, an essential controversy that needs to be thoroughly addressed. Furthermore, the impact of human fungal microbiota on the function of Dectin-1 in tumors is not well studied, and further research is urgently needed.

### The Anti-tumor Role of Dectin-2 and Dectin-3 in Cancers

Dectin-2, encoded by C-type lectin domain family 6 member A (CLEC6A), is generally expressed in myeloid cells, including various subtypes of DCs and macrophages upon activation (Taylor et al., 2005; Robinson et al., 2009). Dectin-2 can bind with α-mannan or zymosan, the key components of *C. albicans, Cryptococcus neoformans*, and others. Dectin-3, also known as Macrophage C-Type Lectin (MCL), encoded by C-type lectin domain family 4 member D (CLEC4D), has a similar structure as Dectin-2 (Ariizumi et al., 2000). Dectin-3 is expressed in peripheral blood neutrophils, monocytes, and various subsets of DCs (Graham et al., 2012). Dectin-3 primarily recognizes trehalose 6, 6-dimycolate (TDM) and α-mannan expressed on the hyphal form of the fungi cell wall (Arce et al., 2004; Graham et al., 2012). Dectin-3 can form a heterodimeric complex with Dectin-2 to recognize α-mannan, which has a higher affinity than homodimers of Dectin-2 or Dectin-3 for sensing *C. albicans* infections and induces a potent activation of NF-κB-dependent anti-fungal immune responses (Zhu et al., 2013).

Recently, Dectin-2 and Dectin-3 in the anti-tumor response have also been studied. Dectin-3 expression was significantly increased in patients with a low fungal burden than in patients with a high fungal burden, indicating the function of Dectin-3 on the host anti-fungal immunity (Zhu et al., 2021). Subsequently, the impact of Dectin-3 on fungal dysbiosis and tumor progression was estimated. Dectin-3 deficiency can promote CAC occurrence, and contribute to the increased fecal fungal burden (Zhu et al., 2021). ITS sequencing showed that the abundance of *C. albicans* was significantly higher in mice lacking the C-type lectin Dectin-3. Further research found the feces of Dectin-3 knockout tumor-bearing mice, as well as *C. albicans*, promote the malignant process of CAC, and anti-fungal treatment (fluconazole) effectively alleviates the tumor load of Dectin-3 knockout mice (Zhu et al., 2021). *In vitro* and *in vivo* experiments demonstrated that Dectin-3 deletion led to impaired clearance of *C. albicans* by macrophages. Increased *C. albicans* induced the upregulation of glycolysis levels in macrophages *via* the HIF-1 pathway, triggering an increased secretion of IL-7 from macrophages. In turn, IL-7 could induce IL-22 production in intestinal intrinsic lymphocytes 3, which ultimately promotes the proliferation of IECs and the progression of CAC (Zhu et al., 2021).

Kimura et al. (2016) also found that Dectin-2 was expressed on resident liver macrophages (Kupffer cells) and played a key role in inhibiting liver metastasis by enhancing the phagocytic activity of macrophages toward colon carcinoma and melanoma cells. Interestingly, this Dectin-2-mediated activity was specific to Kupffer cells, as neither bone marrow-derived macrophages (BMDMs) nor alveolar macrophages engulfed the colon carcinoma in a Dectin-2-dependent manner (Kimura et al., 2016). Furthermore, wild-type mice were treated with antibacterial or anti-fungal antibiotics, but no significant increase was found in colon carcinoma metastasis in the liver, suggesting that Dectin-2 in Kupffer cells acts independently of microbiota on cancer cells (Kimura et al., 2016). Similar to Dectin-2, Dectin-3 also mediates the uptake of colon cancer cells by Kupffer cells and inhibits liver metastasis. Notably, Dectin-1 is also involved in metastatic immune responses in the liver, independent of the phagocytotic activity of Kupffer cells against colon carcinoma cells. Instead, Dectin-1-induced anti-tumor killing was mediated by hepatic non-parenchymal cells (NPCs) (Kimura et al., 2016). As we know, NK cells are primarily responsible for the cytotoxic activity of liver NPCs (Cohen et al., 1990), which is consistent with previous findings (Chiba et al., 2014) that Dectin-1 signaling in DCs and macrophages enhances NK cell-mediated tumoricidal activity.

Current evidence suggests that Dectin-2 and Dectin-3 play an anti-tumor role. Manipulating their anti-tumor response to control CRC formation and liver metastasis is a promising therapeutic approach.

### The Pro-cancer Role of Mincle in Cancers

Mincle, encoded by C-type lectin domain family 4 member E (CLEC4E), also known as Macrophage-Inducible C-Type Lectin, is mainly expressed on professional antigen-presenting cells (APCs), such as macrophages, DCs, B cells, and neutrophils (Yamasaki et al., 2009). Mincle plays a crucial role in anti-fungal immunity, recognizing pathogenic fungi, such as *Malassezia, C. albicans*, and *Fonsecaea pedrosoi* (Wells et al., 2008; Yamasaki et al., 2009; Sousa Mda et al., 2011).

Unlike Dectin-2 and Dectin-3, recent studies have revealed a pro-cancer role for Mincle in the progression of various cancers. The seminal study by Roperto et al. (2015) showed that Mincle expression was increased in urothelial tumors of the urinary bladder of cattle compared to healthy individuals. As we know, the anti-tuberculosis vaccine strain, Bacillus Calmette-Guérin (BCG) is used clinically as an immunotherapy against bladder cancer (Gandhi et al., 2013). The immune mechanism behind this is not well understood (Redelman-Sidi et al., 2014), but BCG-derived TDM might be involved, as Mincle is also a key receptor for TDM (Ishikawa et al., 2009). Li et al. (2020) demonstrated that Mincle was highly expressed in tumor-associated macrophages (TAMs) and significantly associated with poor prognosis in patients with non-small cell lung cancer (NSCLC). The secretome of cancer cells induced Mincle expression in TAMs, and Mincle promoted M2 polarization of TAMs by suppressing the M1 phenotype, thus promoting cancer progression in invasive lung cancer and melanoma. Mechanistic studies revealed that Mincle promoted TAM-mediated pro-tumoral activities *via* the Syk-NF-κB-IL-6 inflammatory pathway (Li et al., 2020). IL-6 is a cancer promoter (Feng et al., 2018), produced mainly by TAMs (Lv et al., 2016), and is thought to be a poor prognostic factor for various cancers (Tang et al., 2018a,b).

Furthermore, Seifert et al. (2016) reported that in PDA, SAP130 (a subunit of the histone deacetylase complex) expression was evident in both epithelial and inflammatory cells. In contrast, Mincle was only highly expressed in inflammatory cells (MDSCs, DCs, and macrophages). Ligation of Mincle by SAP130 increased the infiltration of M2 macrophages and MDSCs and inhibited cytotoxic T-cell function, which induced pancreatic tumorigenesis. Mincle deletion markedly suppressed pancreatic tumorigenesis by enhancing the immunogenicity of TME (Seifert et al., 2016). For treating the carcinogenic effects of Mincle, Xue et al. (2021) successfully developed a novel virus-free gene therapy, USMB-shMincle, by combining RNA interference technology with an ultrasound-microbubble (USMB)-mediated delivery system, and demonstrated its anti-cancer efficiency and safety in two xenograft nude mouse models of human melanoma and lung cancer. USMB-shMincle significantly enhanced the anti-cancer M1 phenotype of TAMs by blocking the protumoral Mincle-Syk-NF-κB-IL-6 signaling. Therefore, Mincle may represent a new target for treating aggressive cancers.

### The Role of Caspase Recruitment Domain-Containing Protein 9 in Cancers

Caspase recruitment domain-containing protein 9 is an intracellular adapter protein from the CARD protein family and is identified for its selective binding to the CARD domain of BCL10 (Bertin et al., 2000). CARD9 is selectively expressed in myeloid-derived innate immune cells, especially in macrophages and dendritic cells (Hsu et al., 2007; Roth and Ruland, 2013), where it operates as a central integrator of the innate and adaptive immune system. It can integrate signals from the CLR and plays a very important role downstream of the anti-fungal Dectin-1, Dectin-2, Dectin-3, and Mincle (Gross et al., 2006; Robinson et al., 2009; Shenderov et al., 2013). Recent studies have confirmed that CARD9 may play a crucial role in a variety of cancers, particularly CRC and lung cancer.

#### The Pro- and Anti-cancer Roles of Caspase Recruitment Domain-Containing Protein 9 in Colorectal Cancer

**FIGURE 4:**
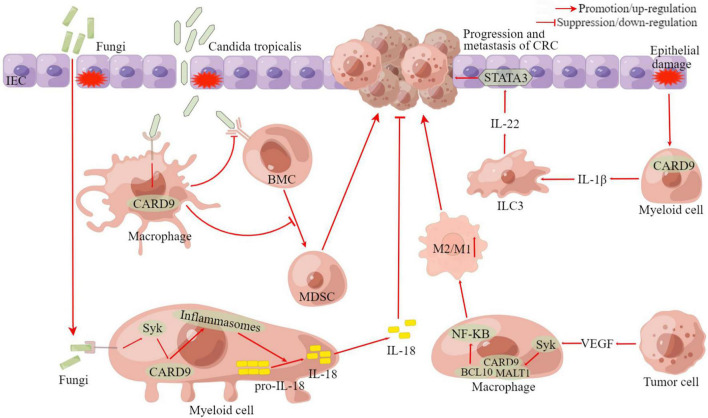
The pro-cancer and anti-cancer roles of CARD9 in CRC. BMC, bone marrow cell; MDSC, myeloid-derived suppressor cell; ILC3, group 3 innate lymphoid cell; CRC, colorectal cancer; VEGF, vascular endothelial growth factor; STAT3, signal transducer and activator of transcription 3; BCL10, B-cell leukemia-lymphoma 10; MALT1, mucosa-associated-lymphoid-tissue lymphoma-translocation gene 1; Syk, Spleen Tyrosine Kinase; IEC, intestinal epithelial cell; CARD9, caspase recruitment domain-containing protein 9; M1/M2, M1 macrophage/M2 macrophage; IL, Interleukin. This figure was created using Figdraw (www.figdraw.com).

Wang T. et al. (2018) divided the patients with CRC into two groups according to their fungal loads and estimated the expression level of CARD9 in tumor tissues. They found that CARD9 expression in the patients with low fungal loads was significantly higher than in the patients with high fungal loads. Moreover, CARD9 knockout (CARD9^–/–^) mice had increased tumor loads and the accumulation of MDSCs in tumor tissue (Wang T. et al., 2018). MDSCs are a heterogeneous group of immature myeloid cells that can promote immune suppression and facilitate tumor development (Gabrilovich and Nagaraj, 2009). The impaired fungicidal functions of CARD9^–/–^ macrophages induced gut mycobiota dysbiosis, with significant enrichment in *Candida tropicalis* (Wang T. et al., 2018). Interestingly, the proportion of *C. tropicalis* was also higher in patients with CRC. The feces of CARD9^–/–^ knockout mice and *C. tropicalis* could promote the development of CAC. Bone marrow cells incubated with *C. tropicalis* exhibited MDSC features and immune suppressive functions (Wang T. et al., 2018). The fluconazole treatment could ameliorate CAC in CARD9^–/–^ mice and is related to decreasing MDSCs accumulation. Thus, after tissue damage induced by dextran sodium sulfate, *C. tropicalis*, translocated into the colonic lamina propria, could be cleared by macrophages through CARD9-dependent antifungal innate immune responses. CARD9^–/–^ mice have impaired fungicidal abilities, which led to increased fungal burden and MDSCs accumulation, inhibition of effector T cells, and the promotion of CRC development (Wang T. et al., 2018; Figure 4). Moreover, the team also recently revealed that *C. tropicalis* induced chemotherapy resistance in CRC *via* increasing lactate production to regulate the mismatch repair system (Qu et al., 2021). Coincidentally, research by Malik et al. (2018) was also published in the same issue of immunity. Malik et al. (2018) found that the intestinal mycobiome promoted inflammasome activation and IL-18 maturation during colitis. Inflammasomes are multimeric protein complexes that form upon sensing a diverse range of danger-associated molecular patterns (DAMPs) and PAMPs (Lamkanfi and Dixit, 2014). Early IL-18 maturation by inflammasome promoted epithelial barrier restitution and interferon-gamma (IFN-γ) production from the CD8^+^ T cells (Malik et al., 2018). After observing that CARD9 expression was increased in human CRC biopsies, they demonstrated myeloid cell-specific deletion of CARD9 or its upstream activator Syk can reduce inflammasome activation and IL-18 maturation and increase its susceptibility to colitis and CAC (Malik et al., 2018). Similarly, treatment with anti-fungal agents (amphotericin B or itraconazole) aggravated colitis and CAC in wild-type mice. Interestingly, a higher tumor burden was also observed in wild-type mice housed with CARD9^–/–^ mice than in wild-type mice alone (Malik et al., 2018). Thus, fungi signaling *via* the Syk-CARD9 axis promotes inflammasome assembly, and subsequent IL-18 maturation upregulates anti-tumorigenic T-cell responses and protects against colitis and CAC (Figure 4).

Although both studies (Malik et al., 2018; Wang T. et al., 2018) employed the same gene-targeted mice, at first glance, it appears to describe somewhat contradictory data. Wang T. et al. (2018) showed that CARD9 was involved in controlling *C. tropicalis* replication in macrophages. *C. tropicalis* can induce differentiation of MDSCs with immunosuppressive effects, and monocultures of *C. tropicalis* in germ-free mice can enhance tumor load, suggesting the pro-tumorigenic properties of this fungus. In contrast, Malik et al. (2018) implied that fungal-induced CARD9 activation inhibited tumor growth by regulating IL-18 release, indicating that commensal fungi may be responsible for tumor suppressor signaling events. Despite the discrepancies, there is no denying that both articles provide strong support for a decisive role of mycobiota in CAC, a role that has hitherto been underestimated.

In addition to studies by Malik et al. (2018) and Wang T. et al. (2018), which concluded that CARD9 is an anti-tumor molecule in the development of CAC, more studies have found that CARD9 plays a pro-cancer role in CRC using CARD9^–/–^ mice (Yang et al., 2014; Leo et al., 2015; Bergmann et al., 2017). Bergmann et al. (2017) found that CARD9-signaling drove the generation of IL-1β by myeloid cells within the damaged intestine and regulated the subsequent production of IL-22 by group 3 innate lymphoid cells (ILC3), which ultimately promoted tumorigenesis *via* signal transducer and activator of transcription 3 (STAT3) activation within the transformed epithelium (Figure 4). Another study found that CARD9 was significantly more expressed in colon cancer tissues compared to adjacent normal tissues, primarily in tumor-infiltrating leukocytes but not cancer cells (Yang et al., 2014). The expression levels of CARD9 correlated positively with the advanced histopathological stage and the presence of metastasis and recurrence. In TME, tumor cell-secreted vascular endothelial growth factor (VEGF) facilitated the activation of the Syk signaling pathway in macrophages, which induced the assembly of CARD9-BCL10-MALT1 complexes (Yang et al., 2014). Subsequently, CARD9 initiated macrophage to M2 macrophage polarization through the activation of the NF-κB pathway, contributing to tumor metastasis (Yang et al., 2014; Figure 4). The well-known APC^min^ mouse model is used to mimic human familial adenomatous polyposis (Kwong and Dove, 2009). CARD9 was found to reduce the viability specifically in males and promote tumorigenesis in the large intestines of these male APC^min^ mice (Leo et al., 2015).

#### The Pro- and Anti-cancer Roles of Caspase Recruitment Domain-Containing Protein 9 in Lung Cancer

The role of CARD9 in lung cancer is also controversial. Miwa et al. (2020) found that CARD9 was expressed in the cytoplasm of tumor cells. The high expression was associated with poorer OS compared to the low expression of CARD9 (Miwa et al., 2020). CARD9 activated the NF-κB pathway in tumor cell lines, and the knockdown of CARD9 inhibited proliferation in tumor cells (Miwa et al., 2020). However, there is growing evidence that CARD9 appears to play an anti-cancer role in lung cancer. It was demonstrated that CARD9 expression was reduced in NSCLC tissues compared to normal tissues, and low CARD9 expression was related to poor survival (Pan et al., 2020). CARD9 was expressed in both tumor cells and macrophages, and the downregulation of CARD9 in NSCLC cells enhanced proliferation, invasion, and migration through the activation of MAPK/p38 signaling, while the overexpression of CARD9 presented anti-tumor effects. Another study revealed that CARD9 was predominantly expressed in myeloid cells in lung cancer (Qu et al., 2019). Tumor tissues from CARD9^–/–^ mice with lung cancer were found to have more MDSCs, less cytotoxic T lymphocytes, and a heavier tumor burden than wild-type mice. In contrast, depletion of MDSCs significantly reduced the tumor burden in CARD9^–/–^ tumor-bearing mice (Qu et al., 2019). Further studies suggested that in MDSCs, CARD9 inhibited the production of indoleamine 2,3-dioxygenase (IDO), an immunosuppressive enzyme produced by MDSCs, by suppressing the non-canonical NF-κB pathway, ultimately leading to the functional limitation of MDSCs and preventing the development of lung cancer (Qu et al., 2019; Wu et al., 2019). Additionally, Wang et al. (2020) demonstrated that Ganoderma lucidum polysaccharide strengthened anti-tumor immune responses by regulating the differentiation and inhibition of MDSCs *via* a CARD9- canonical NF-κB-IDO pathway. In summary, the current evidence seems to favor an anti-cancer role for CARD9 in lung cancer.

#### The Pro-cancer Role of Caspase Recruitment Domain-Containing Protein 9 in Other Cancers

The oncogenic role of CARD9 has been identified in other tumors. As early as 2005, it was found that the overexpression of CARD9 might be associated with the development or progression of gastric lymphoma, particularly in patients whose pathogenesis is unrelated to *H. pylori* (Nakamura et al., 2005; Zhou et al., 2006). Subsequently, it was confirmed that high expression of CARD9 was significantly related to a 2.52-fold increased risk of death in ccRCC (Tan et al., 2011). The von Hippel-Lindau tumor suppressor protein (pVHL) was found to promote the inhibitory phosphorylation of CARD9 by Casein Kinase 2 (CK2) (Yang et al., 2007). The pVHL deficiency increased c-Jun N-terminal kinase (JNK) and NF-κB activity due to overactive CARD9, leading to proapoptotic cytokine resistance and uncontrolled growth of RCC (Yang et al., 2007; An et al., 2013). CARD9 is also upregulated in tissues of OSCC, and CARD9 expression is strongly associated with poor differentiation degree, III-IV TNM stage, and lymphatic metastasis (Ye et al., 2019). While CARD9 was inhibited, the activity of the NF-κB signaling pathway was weakened, with decreases in the proliferation, invasion, and migration of OSCC cells, but enhanced apoptosis (Ye et al., 2019). Similar findings have been found in ESCC; high CARD9 expression was significantly associated with advanced tumor depth, positive lymph node metastases, and advanced stage (Sekino et al., 2020). OS and disease-free survival were significantly shorter in patients with ESCC patients who have high CARD9 expression (Sekino et al., 2020). Knockdown of CARD9 resulted in a significant reduction in the proliferation and migration capacity of ESCC cell lines (Sekino et al., 2020). Moreover, in high-grade serous ovarian cancer, Rad50 activates the NF-κB pathway through interaction with CARD9, which induces tumor cell proliferation, invasion, and epithelial-mesenchymal transition (Li et al., 2021).

Additionally, CARD9 has been suggested to be used to diagnose tumors. Zekri et al. (2013) studied 130 patients with hepatitis C virus (HCV)-associated liver disease, 40 of whom were diagnosed with HCC. The results showed that the expression level of CARD9 in HCC was significantly higher than in chronic HCV, chronic active hepatitis, and cirrhosis, and could be a marker for HCC diagnosis and a candidate gene for molecularly targeted therapy (Zekri et al., 2013). Furthermore, Li et al. (2016) found that compared to benign pleural effusion, CARD9 was significantly reduced in malignant pleural effusion and could serve as a helpful peptide biomarker for diagnosing malignant pleural effusion (Li et al., 2016).

In conclusion, as an important adapter molecule in fungal immunity, CARD9 exhibits pro- and anti-cancer properties. The role played by the fungus in this process is not fully understood and deserves further investigation. Moreover, CARD9 is also expressed on tumor cells. However, it is yet to be understood whether tumor cells have a function in recognizing fungi.

## Conclusion and Future Perspective

Current evidence reveals an association between gut mycobiota and the development of many human cancers. The fungal recognition receptors (Dectin-1, Dectin-2, Dectin-3, and Mincle) play a pro- or anti-cancer role in TME, depending on the context. This study demonstrates that fungal pathogens may induce inflammatory responses, contributing to tumorigenesis. Could these immune responses to fungi put other organs at the risk of developing cancer? As our understanding of the seemingly plausible relationship between human fungi and cancer expands, emerging information will shed light on this intriguing issue. In addition, more studies are needed to fully characterize the human gut microbiome. Inter-kingdom interactions between the human bacteriome and mycobiome might unlock new pathways that could explain many unanswered questions. At the same time, multi-omics studies are mandatory in the daunting task of finding potential biomarkers and therapeutic targets for cancer and the human mycobiome. Large-scale, long-term, prospective, and longitudinal studies using multi-omics approaches are needed in the future to examine the role of the fungi in cancer pathogenesis and to determine whether changes that occur in the mycobiome are causal or a consequence of cancer.

## Author Contributions

LZ, DC, CC, WD, and WW conceived and designed the study. LZ, DC, CC, CL, ZQ, and TK searched the literature and wrote the manuscript. LZ, DC, CC, MP, KD, JY, WD, and WW revised the manuscript. All the work was performed under the instruction of WD and WW. All authors read and approved the final manuscript.

## Conflict of Interest

The authors declare that the research was conducted in the absence of any commercial or financial relationships that could be construed as a potential conflict of interest.

## Publisher’s Note

All claims expressed in this article are solely those of the authors and do not necessarily represent those of their affiliated organizations, or those of the publisher, the editors and the reviewers. Any product that may be evaluated in this article, or claim that may be made by its manufacturer, is not guaranteed or endorsed by the publisher.
